# Expression profile of RNA binding protein in cervical cancer using bioinformatics approach

**DOI:** 10.1186/s12935-021-02319-7

**Published:** 2021-12-04

**Authors:** Zhiyuan Huang, Fang Li, Qinchuan Li

**Affiliations:** 1 Research Center for Translational Medicine, Shanghai East Hospital, School of Medicine, Tongji University, Shanghai, 200120 China; 2 Department of Gynecology, Shanghai East Hospital, School of Medicine, Tongji University, Shanghai, 200120 China; 3 Department of Cardiothoracic Surgery, Shanghai East Hospital, School of Medicine, Tongji University, Shanghai, 200120 China

**Keywords:** RNA binding proteins, Cervical cancer, Prognosis, TCGA, GTEx

## Abstract

**Background:**

It has been demonstrated by studies globally that RNA binding proteins (RBPs) took part in the development of cervical cancer (CC). Few studies concentrated on the correlation between RBPs and overall survival of CC patients. We retrieved significant DEGs (differently expressed genes, RNA binding proteins) correlated to the process of cervical cancer development.

**Methods:**

Expressions level of genes in cervical cancer and normal tissue samples were obtained from GTEx and TCGA database. Differently expressed RNA binding proteins (DEGs) were retrieved by Wilcoxon sum-rank test. ClusterProfiler package worked in R software was used to perform GO and KEGG enrichment analyses. Univariate proportional hazard cox regression and multivariate proportional hazard cox regressions were applied to identify DEGs equipped with prognostic value and other clinical independent risk factors. ROC curve was drawn for comparing the survival predict feasibility of risk score with other risk factors in CC patients. Nomogram was drawn to exhibit the prediction model and validated by C-index and calibration curve. Correlations between differentially expressed RNA binding proteins (DEGs) and other clinical features were investigated by t test or Cruskal Wallis analysis. Correlation between Immune and DEGs in cervical cancer was investigated by ssGSEA.

**Results:**

347 differentially expressed RBPs (DEGs) were retrieved from cervical cancer tissue and normal tissue samples. GO enrichment analysis showed that these DEGs involved in RNA splicing, catabolic process and metabolism. Cox regression model showed that there were ten DEGs significantly associated with overall survival of cervical cancer patients. WDR43 (HR = 0.423, P = 0.008), RBM38 (HR = 0.533, P < 0.001), RNASEH2A (HR = 0.474, P = 0.002) and HENMT1 (HR = 0.720, P = 0.071) played protective roles in survival among these ten genes. Stage (Stage IV vs Stage I HR = 3.434, P < 0.001) and risk score (HR = 1.214, P < 0.001) were sorted as independent prognostic risk factors based on multivariate cox regression. ROC curve validated that risk score was preferable to predict survival of CC patients than other risk factors. Additionally, we found some of these ten predictor DEGs were correlated significantly in statistic with tumor grade or stage, clinical T stage, clinical N stage, pathology or risk score (all P < 0.05). Part of immune cells and immune functions showed a lower activity in high risk group than low risk group which is stratified by median risk score.

**Conclusion:**

Our discovery showed that many RNA binding proteins involved in the progress of cervical cancer, which could probably serve as prognostic biomarkers and accelerate the discovery of treatment targets for CC patients.

**Supplementary Information:**

The online version contains supplementary material available at 10.1186/s12935-021-02319-7.

## Introduction

One of the most challenging malignancies is cervical cancer (CC) observing among females worldwide [1]. It is showed that CC led to more than about 311 thousand people death all over the world in statistically in 2018 [2]. One of major reasons for cervical cancer is infection of high-risk human papillomavirus (HPV), although the occurrence of cervical cancer cannot be fully elucidated by HPV infection [3]. Radical hysterectomy, radical radiotherapy and chemotherapy based on cisplatin are major treatment methods for CC patients until now [4]. It is reported that patients with locally advanced cervical cancer had their 5-year overall survival (OS) increased into about 70% after chemotherapy [5]. Nonetheless, the recurrence of cervical cancer after surgery or radiotherapy remains a problem difficult to solve [6]. The circumstance of limited treatment and a poor prognosis is the reality that CC patients with relapse have to face [7, 8].

RNA binding proteins (RBPs) belong to one of the crucial series cellular proteins. Their interaction with RNA by means of recognizing special RNA binding domains plays a significant role in varies kinds of post-transcriptional regulation. For example, RNA transport, translation control, intracellular localization, shearing, sequence editing are both under the influence of RNA binding proteins [9]. Former studies have discovered more than 1500 proteins who involved in RNA binding in *Homo sapiens* genome [10]. There is a significant district in RBPs, which contains 60–100 residues. This district often adopts an αβ topology which assists them to bind the RNA according to concrete nucleic acid sequence [11]. The origination and development of many diseases have been discovered to be correlated with RBPs. For example, spinal muscular atrophy and myotonic dystrophy are two kinds of typical disease [12]. Undoubtedly, the origination and development of cancer has been reported to have something to do with RBPs. For example, HuR, which is a RBP is able to accelerate the proliferation and promote metastasis of gastric cancer [13]. Zhang et al. reported that AGO2 increases oncogenic miR-19b biogenesis by Acetylation which leads to the facilitation of cancer progression [14]. The proliferation of lung cancer cells can be regulated by cancer-associated alternative splicing. This process is inhibited by QKI-5 [15]. ESRP1 accelerates the EMT of ovarian carcinoma cells [16]. All these researches revealed RBPs as important adjustment molecules in the process of cancer development.

Nowadays, FIGO stage serves as a majority tool for doctor to predict the survival of cervical cancer patients in clinical [17]. There is deficiency in FIGO stage system that patients may have different individual survival time even if they are attributed to same FIGO stage [18]. In order to provide doctors with a better prognostic prediction tool for CC patients, more clinical factors should be taken into consideration. Recently, the prognosis model involved with the expression level of RBPs has become popular and been constructed in colorectal cancer [19], hepatocellular cancer [20] and breast cancer [21], etc. So, the prognostic prediction role of RNA binding proteins in CC trigged our interest. To begin with, the differently expressed RNA binding proteins (DEGs, differently expressed genes) were retrieved from gene expression profile of tumor tissues and normal tissues. They were uploaded to STRING database for constructing protein–protein interaction network. A cox regression model for predicting the survival of cervical cancer patients was constructed with DEGs involved in the PPI network prognosis signature. The predict factors involved in this cox regression model had been validated by the Kaplan–Meier analysis. The survival status discrimination efficacy of risk score was compared with other clinical factors by means of ROC curve and quantified by area under the curve (AUC). Moreover, GO and KEGG enrichment analysis was applied to explore the functional pathways that screened DEGs in PPI network and their subnetworks involved in. Finally, we also explored the relationships between the risk scores which was counted by DEGs signature and immune cells or functions.

## Materials and methods

### Acquisition data from GTEx and TCGA dataset

The expression level of genes in normal cervix was downloaded from GTEx database (https://www.genome.gov/Funded-Programs-Projects/Genotype-Tissue-Expression-Project), which is a website containing great quantity of gene expression data resourced from healthy people. The expression level of genes in cervical cancer and the corresponding clinical data were downloaded from TCGA database (https://portal.gdc.cancer.gov/), which is a landmark cancer genomics program. Clinical pathological data of patients from TCGA is available in Additional file 1: Table S1. Gene array data from GTEx and TCGA was normalized by means of limma package from R bioconductor software. Totally 1542 RBPs (RNA binding proteins) [10] were obtained to screen the gene expression profile.

### Identification of DEGs (different expression genes, different expression RBPs)

Wilcoxson signed-rank test was applied to identify differentially expressed RNA binding protein genes (DEGs). The cut-off values were set based on left parameters, false discovery rate (FDR) < 0.05 and |log2 fold change (logFC)|> 0.5.

### Functional enrichment analysis

Gene Ontology (GO) and Kyoto Encyclopedia of Genes and Genomes (KEGG) pathway enrichment analysis were performed by clusterProfiler package [22] in R software. Results was filtered by FDR (false discovery rate) < 0.05, top result were presented and recognized as significant items.

### Construct protein–protein interaction network and the subnetwork

The sorted DEGs were applied to construct protein–protein interaction network by STRING (The Search Tool for the Retrieval of Interacting Genes). The PPI network was visualized by Cytoscape software. The MCODE which is a plug-in for Cytoscape was applied to obtain the first three relevant sub-network modules. The genes in them were applied to perform GO and KEGG enrichment analysis respectively.

### Construct cox regression model

The correlation between the overall survival (OS) and differently expressed RNA binding proteins (DEGs) was firstly investigated by univariate cox regression. Variables were screened by p-value (< 0.05) presented from Wald X^2^ test. All the significant variables were applied to construct multivariate cox regression model and sorted by AIC value. Then, the expression level of significant DEGs were included into multivariate cox regression with other clinical factors to construct prognostic predict model.

### ROC curve analysis

The prediction value of independent risk factors was investigated by Receiver Operating Characteristic (ROC) curve with area under the curve (AUC). ROC curve was draw by survival ROC package in R software, which is designed for survival data. AUC was applied to measure the sensitivity and specificity of prediction variable, which ranges from 0.5 to 1. The larger the AUC is, the better the variable predict the prognosis.

### Development of the nomogram

Nomogram was drawn to exhibit the prediction model constructed based on independent prognostic factors sorted by univariate and multivariate cox regression. In order to evaluate the calibration and discrimination of nomogram, calibration curves were plotted and Harrell C-index was calculated. Bootstrapping validation with 100 bootstraps resample was applied to calculate C-index for this nomogram.

### Immune and RNA binding protein in cervical cancer

The infiltrating scores of 16 immune cells was measured by the single-sample gene set enrichment analysis (ssGSEA) in the "gsva" R package. The activities of 13 immune-related pathways was evaluated in the same way [23]. The annotated gene set file applied in ssGSEA analysis is provided in Additional file 2: Table S2 [24]. TIMER-2 database (http://timer.cistrome.org/) was applied to explore the correlation between prognostic significant RBP genes and six kinds of immune cells (T cell CD8+ , T cell CD4+ , B cell, Neutrophil, Macrophage, Myeloid dendritic).

### Experimental validation by RT-qPCR

RT-qPCR experiment was conducted to validate the expression level of five differentially expressed RNA binding proteins retrieved by bioinformatics analysis. CC specimens and correspondent normal cervical tissues come from CC patients who received operation from 2020 to 2021 January in Shanghai east hospital, school of medicine, Tongji University. The clinical pathological data of patients is available in Additional file 3: Table S3. Internal review board of Shanghai East Hospital, school of medicine, Tongji University have approved this study.

Total RNA was retrieved by TRIzol (invitrogen, USA) from CC tissues and correspondent normal tissues. The purified RNA was transcribed into cDNA (Complementary DNA) by PrimeScript® RT reagent Kit with gDNA (genomic DNA) Eraser (Takara). SYBR master mix kit (vazyme, China) was used to detect the expression level of these DEGs (Differently expressed genes, RNA binding proteins) on the QuantStudio RT-qPCR System (Q6, Applied Biosystems, USA). Endogenous GAPDH (gyceral-dehyde-3-phosphate dehydrogenase) or ACTB (beta actin) was used to normalize the expression level of each genes by 2^−△△Ct^. In order to display whether the expression level of gene is up-regulated or down-regulated in tumor tissues comparing with corresponding normal tissues, the relative expression level of each gene was log2 transformed for barplot. The primers were synthesized by Sangon company and Genscript company, China.

### Kaplan–Meier analysis of prognostic significant RBPs

The patients suffered from cervical cancer were divided into high expression group and low expression group by means of expression level of each prognostic significant RBPs. The survival curves of high expression group and low expression group were drawn by survival package in R software. Their overall survivals were compared by log-rank test.

### OncoPrint evaluation of sorted RBPs in cervical cancer by cBioPortal

In order to explore the gene expression variation of prognosis significant RNA binding proteins across cervical cancer tissues from patients respectively. cBioPortal OncoPrint (http://cbioportal.org, accessed on 10 OCT 2021) was applied to draw a graphical summary, in which 278 cervical cancer samples from patients in TCGA database (TCGA, PanCancer Atlas) involved. In integrated graph, cervical cancer patients were represented as columns, prognosis significant RNA binding proteins were represented as rows. Colour codes and glyphs stood for genomic alterations such as missense mutation, truncating mutation, amplification or deep deletion.

## Results

### Sort DEGs from RNA binding protein gene expression profile

**Fig. 1:**
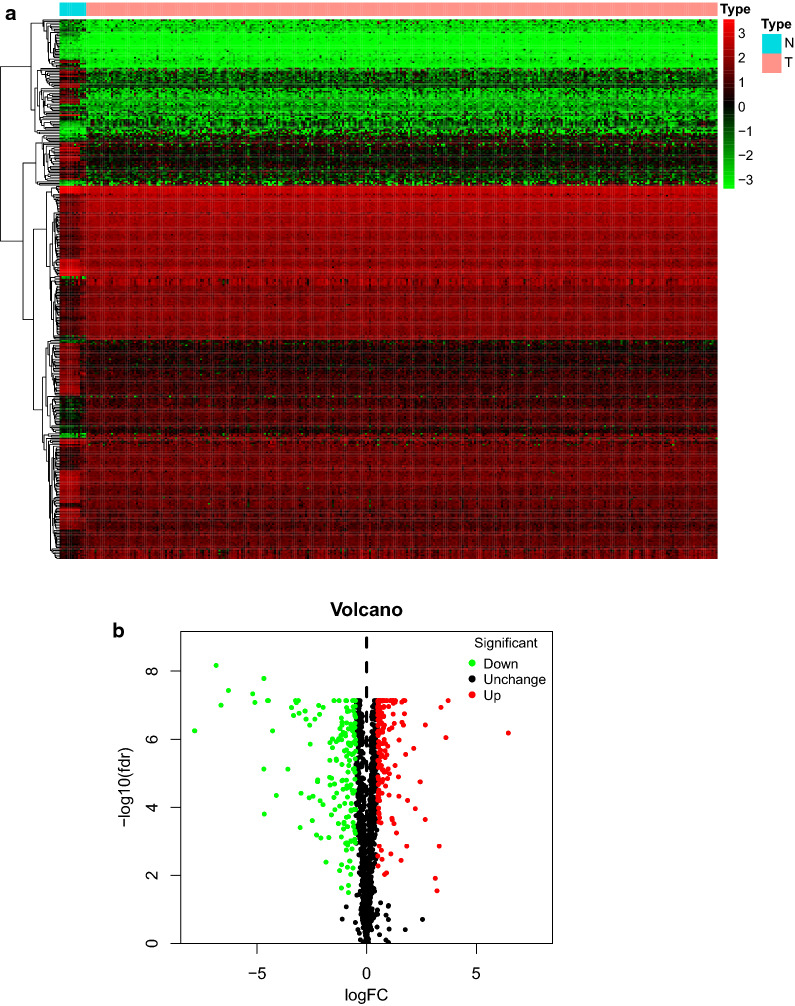
Expression level of DEGs (RNA binding proteins) between normal group and tumor group. **a** Expression levels of DEGs presented in heat map. Down-regulated genes are presented in green and up-regulated genes are presented in red. **b** Expression levels and expression fold changes of DEGs presented in volcano plot. 176 down-regulated genes are presented as green dots; 171 up-regulated genes are presented as red dots

An expression profile dataset was combined with data from GTEx and TCGA database, which included 13 normal cervix samples and 306 cervical cancer samples. The clinical data of the patients was downloaded from TCGA database and integrated into expression matrix by Perl software. We obtained 347 differently expressed RNA bind proteins (DEGs) by comparison of the expression level of RNA bind proteins (DEGs) between cervix tissues and cervical cancer tissues. There were 177 genes down-regulated in tumor samples and 170 genes up-regulated in tumor samples compared with normal samples (Additional file 4: Table S4). The detail of DEGs’ expression matrix was presented by heat map and volcano plot (Fig. 1a, b).

### Bio-functional enrichment analysis of DEGs

We conducted GO and KEGG enrichment analysis by clusterprofiler package in R software to evaluate the biological function of our retrieved DEGs. These differently expressed RBPs were divided into up-regulated group and down-regulated group for enrichment analysis individually.

**Fig. 2:**
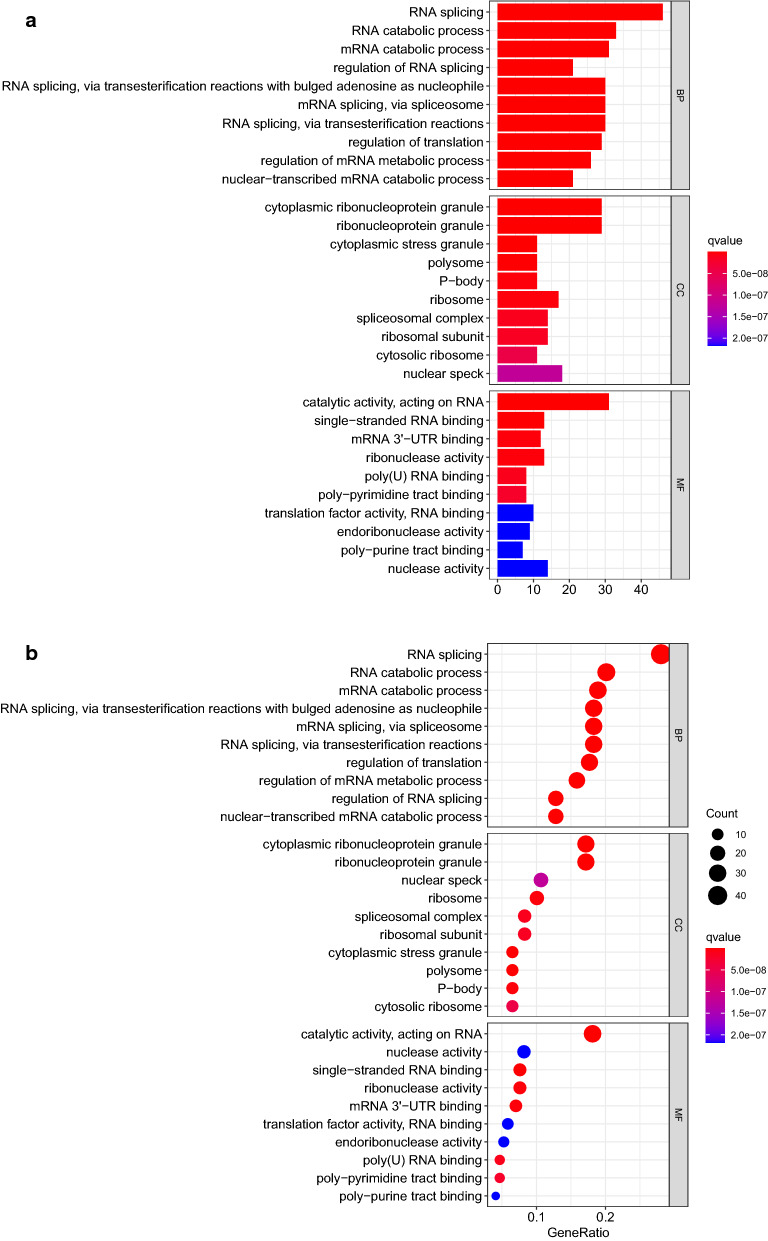

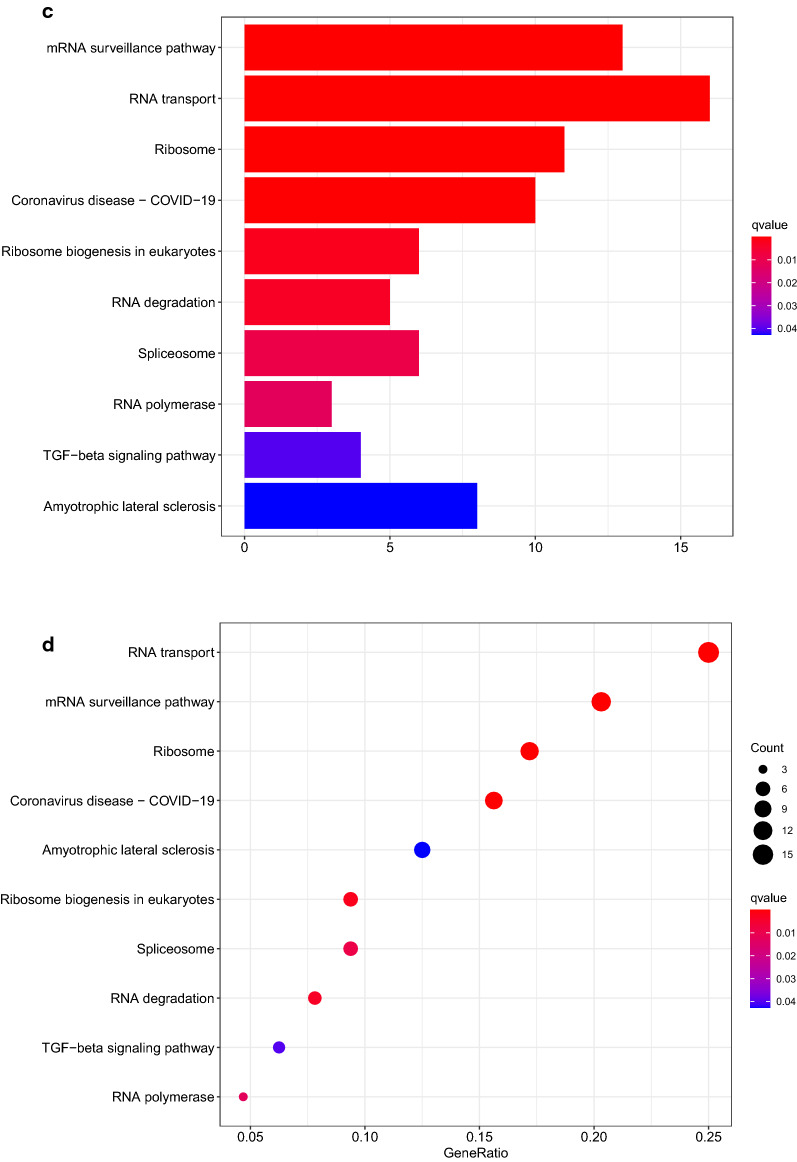
Down regulated DEGs were applied in GO and KEGG enrichment analysis. **a**, **b** GO enrichment analysis were shown in bubbles plot and bar blot respectively. **c**, **d** KEGG enrichment analysis were shown in bubbles plot and bar blot respectively. The significant degree of enrichment was measured by size of bubble and depth of color

In GO analysis, for BP (biological process) category, downregulated DEGs mainly enriched in RNA splicing, RNA catabolic process and mRNA catabolic process. For CC (cellular components) category, downregulated DEGs mainly enriched in cytoplasmic ribonucleoprotein granule, ribonucleoprotein granule and cytoplasmic stress granule. For MF (molecular function) category, downregulated DEGs mainly enriched in catalytic activity, acting on RNA, single-stranded RNA binding and mRNA 3'-UTR binding. In KEGG pathway analysis, down-regulated DEGs mainly enriched in mRNA surveillance pathway, RNA transport and Ribosome (Fig. 2a–d) (Additional file 5: Table S5, Additional file 6: Table S6).

**Fig. 3:**
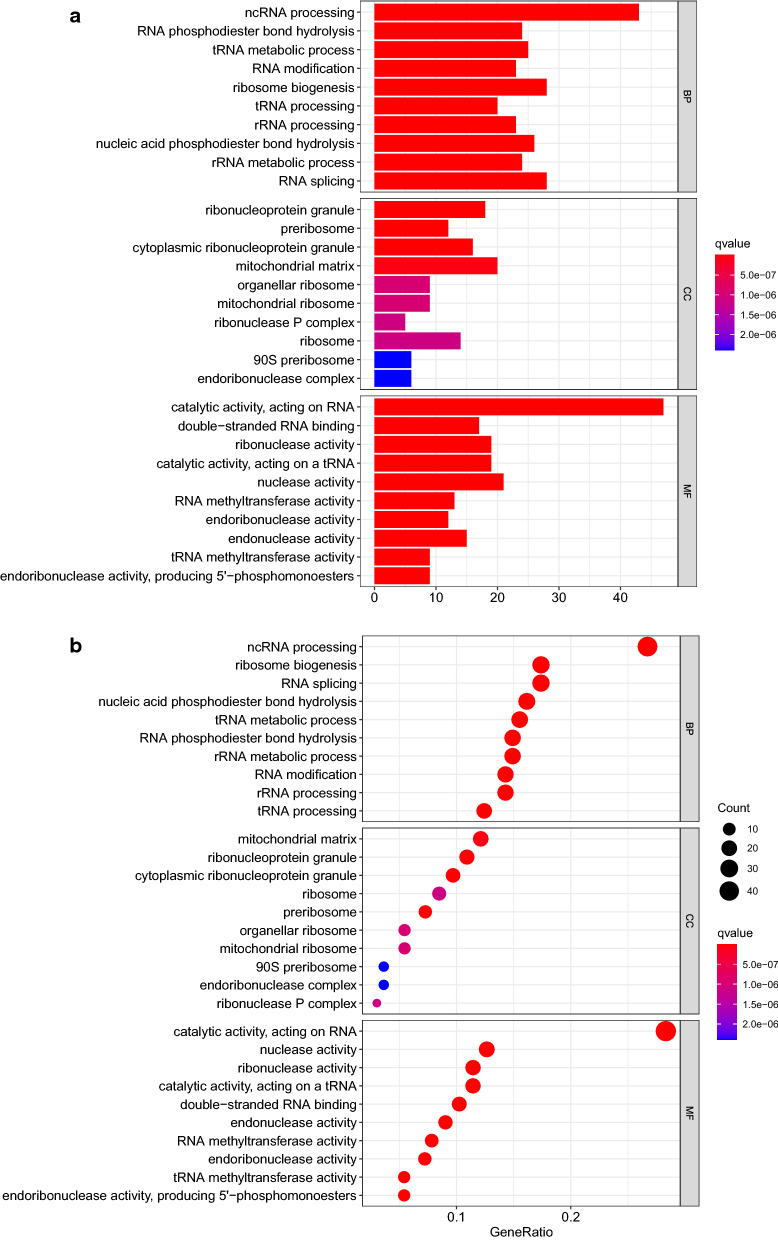

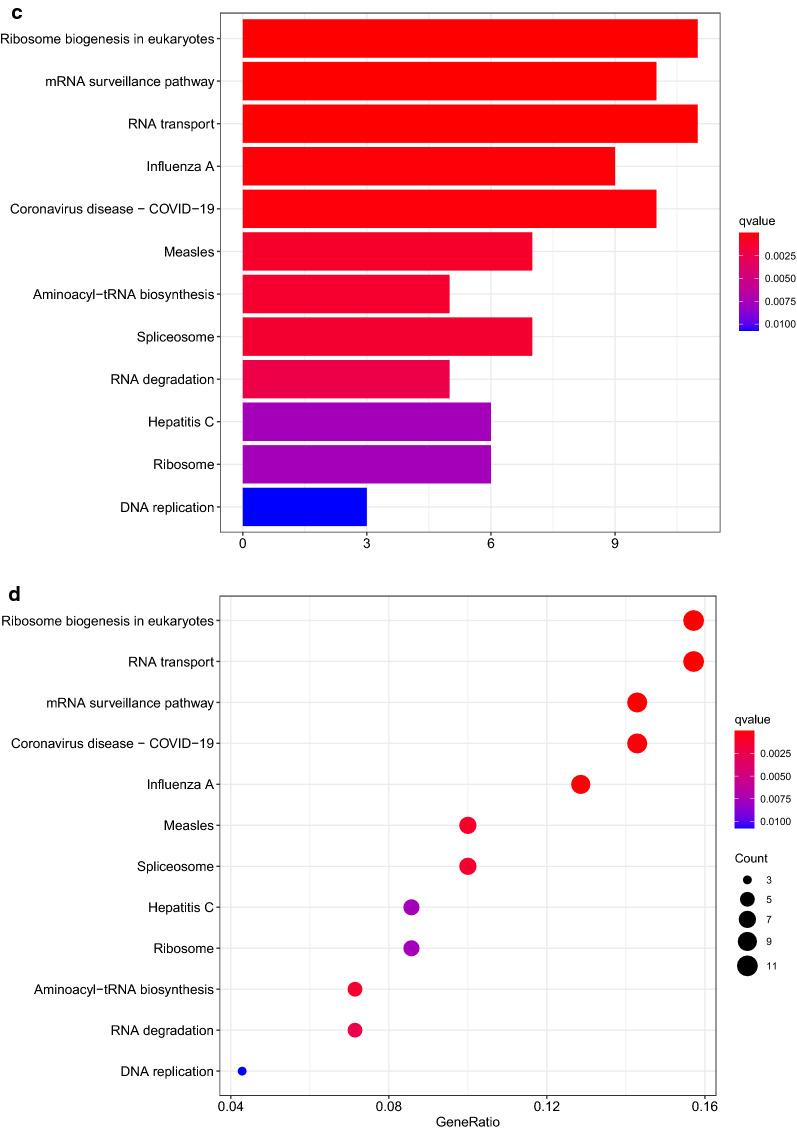
UP regulated DEGs were applied in GO and KEGG enrichment analysis. **a**, **b** GO enrichment analysis were shown in bubbles plot and bar blot respectively. **c**, **d** KEGG enrichment analysis were shown in bubbles plot and bar blot respectively. The significant degree of enrichment was measured by size of bubble and depth of color

In GO analysis for up regulated group, biological process (BP) category mainly includes ncRNA processing, RNA phosphodiester bond hydrolysis, and tRNA metabolic process. They have something to do with the process of cervical cancer. Items in cellular component (CC) mainly include ribonucleoprotein granule and preribosome. They involved in RNA binding and protein expression level adjustment, which may help cervical cancer cells survive. Molecular function (MF) category showed that DEGs were mainly enriched in catalytic activity, acting on RNA, double-stranded RNA binding and ribonuclease activity, respectively. They revealed the function of RBP. In result of KEGG analysis for upregulated DEGs, DEGs were enriched in pathways such as Ribosome biogenesis in eukaryotes, mRNA surveillance pathway and RNA transport. These pathways help cancer cells live a better life (Fig. 3a–d) (Additional file 7: Table S7, Additional file 8: Table S8).

### Construct Protein–protein interaction (PPI) network

**Fig. 4:**
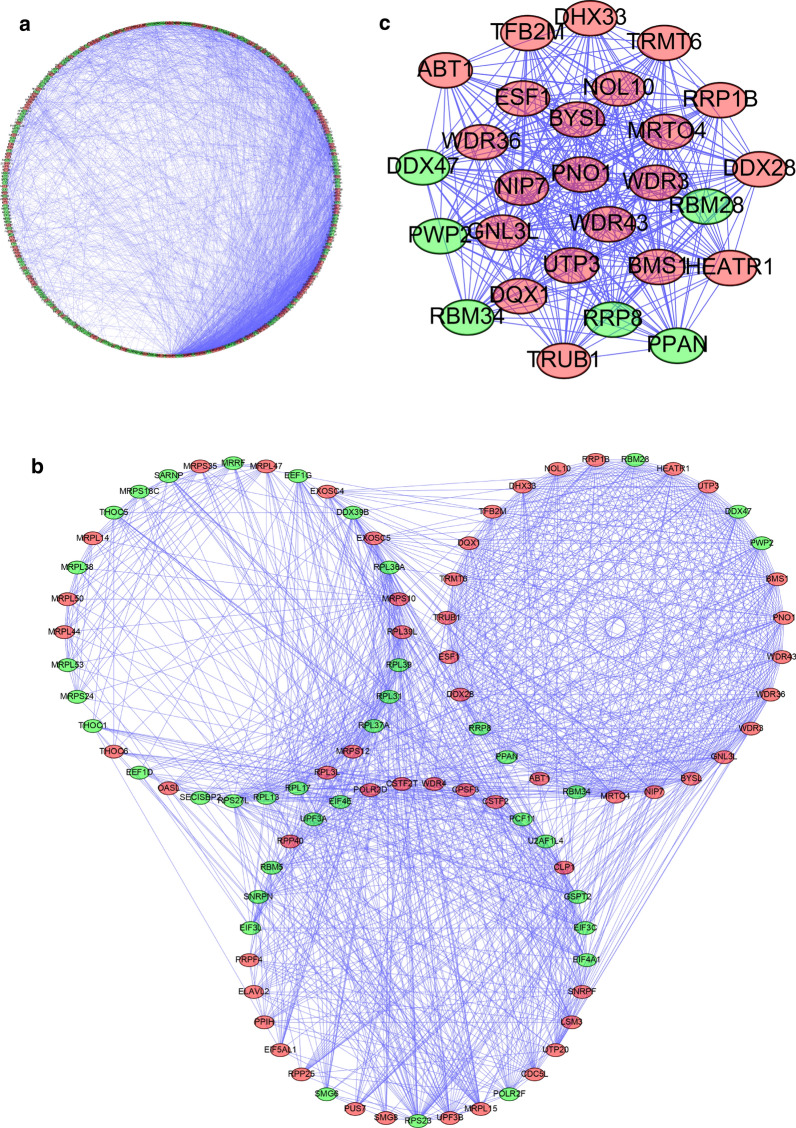
Construct PPI (Protein–protein interaction) network. **a** PPI network of differentially expressed RBPs; RBPs were arranged in a circle. **b** MCODE plug-in sorted three most critical modules from PPI network; RBPs were arranged in three circles. Green circles stood for down-regulated RBPs in CC with a fold change of more than 1.41; Red circles stood for up-regulated RBPs with fold change more than 1.41

A PPI network was constructed by Cytoscape software. The information of nodes and network was obtained from STRING database according to uploaded DEGs. The PPI network incorporated 2545 edges and 320 nodes (Fig. 4a). The co-expression network was treated by MCODE plug-in for Cytoscape to identify the most correlated three subnetworks (Fig. 4b). Acquired first important crucial module consist of 27 nodes and 335 edges (Fig. 4c). The GO enrichment analysis result shows that the RBPs in the key module 1 were mainly enriched in ribosome biogenesis, preribosome and RNA helicase activity. Moreover, in KEGG analysis they were enriched in Ribosome biogenesis in eukaryotes pathway. The GO and KEGG analysis results of both three subnetworks were displayed in Tables 1 and 2.

**Table 1:** The GO enrichment analysis of three most significant MCODE components

Ontology	ID	Description	p value	q value	Count
Subnetwork1					
BP	GO:0042254	Ribosome biogenesis	2.8E−32	3.83E−30	20
BP	GO:0006364	rRNA processing	9.39E−26	5.61E−24	16
BP	GO:0034470	ncRNA processing	1.23E−25	5.61E−24	18
CC	GO:0030684	Preribosome	7.18E−23	8.31E−22	12
CC	GO:0032040	Small-subunit processome	9.73E−12	5.63E−11	6
CC	GO:0030686	90S preribosome	5.39E−10	2.08E−09	5
MF	GO:0003724	RNA helicase activity	5.67E−06	0.000137	4
MF	GO:0140098	Catalytic activity, acting on RNA	2.08E−05	0.000252	6
MF	GO:0004386	Helicase activity	0.000103	0.000833	4
Subnetwork2					
BP	GO:0006414	Translational elongation	1.82E−22	4.15E−20	14
BP	GO:0070126	Mitochondrial translational termination	1.33E−20	1.52E−18	12
BP	GO:0006415	Translational termination	9.55E−20	7.27E−18	12
CC	GO:0044391	Ribosomal subunit	3.49E−33	5.15E−32	20
CC	GO:0005840	Ribosome	5.99E−30	4.42E−29	20
CC	GO:0015934	Large ribosomal subunit	2.13E−23	1.04E−22	14
MF	GO:0003735	Structural constituent of ribosome	5.74E−22	1.27E−20	15
MF	GO:0003746	Translation elongation factor activity	0.000518	0.005724	2
MF	GO:0004540	Ribonuclease activity	0.000931	0.006861	3
Subnetwork3					
BP	GO:0000377	RNA splicing, via transesterification reactions with bulged adenosine as nucleophile	1.03E−20	5.72E−19	17
BP	GO:0000398	mRNA splicing, via spliceosome	1.03E−20	5.72E−19	17
BP	GO:0000375	RNA splicing, via transesterification reactions	1.17E−20	5.72E−19	17
CC	GO:0005849	mRNA cleavage factor complex	1.46E−10	4.75E−09	5
CC	GO:0005681	Spliceosomal complex	5.86E−10	9.57E−09	8
CC	GO:0046540	U4/U6 x U5 tri-snRNP complex	7.79E−09	6.35E−08	5
MF	GO:0008135	Translation factor activity, RNA binding	1.02E−08	4.31E−07	6
MF	GO:0140098	Catalytic activity, acting on RNA	4.10E−07	8.64E−06	8
MF	GO:0003743	Translation initiation factor activity	2.35E−06	3.30E−05	4

**Table 2:** The KEGG pathway analysis of three most significant MCODE components

ID	Description	p value	q value	Count
Subnetwork1				
hsa03008	Ribosome biogenesis in eukaryotes	7.88E−15	8.29E−15	8
Subnetwork2				
hsa03010	Ribosome	2.16E−16	9.10E−16	12
hsa05171	Coronavirus disease—COVID-19	3.72E−08	7.83E−08	8
hsa03013	RNA transport	0.000973	0.001365	4
hsa03018	RNA degradation	0.015887	0.016723	2
Subnetwork3				
hsa03015	mRNA surveillance pathway	4.25E−12	5.37E−11	9
hsa03013	RNA transport	7.41E−07	4.68E−06	7
hsa03040	Spliceosome	3.70E−06	1.56E−05	6
hsa03020	RNA polymerase	0.003703	0.011693	2

### Retrieve DEGs related to prognosis

**Fig. 5:**
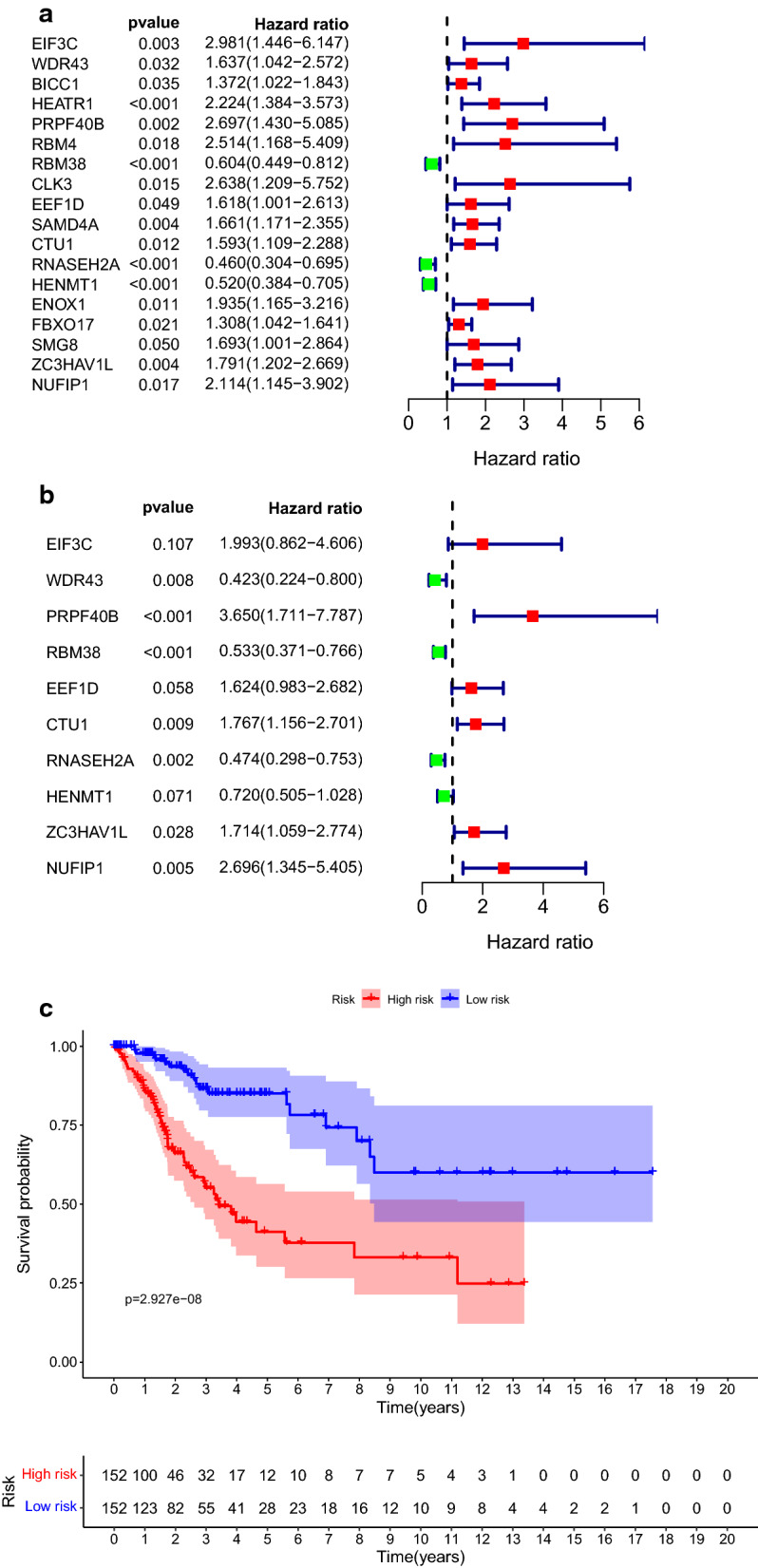
Forest plot of HR of DEGs and Kaplan–Meier curve for DEGs. **a** Forest plot of 18 prognosis-related DEGs retrieved by univariate cox regression. **b** Forest plot of 10 prognosis-related genes retrieved by multivariate cox regression model with AIC value. **c** KM curve for overall survival in the high-risk and the low-risk groups stratified by DEGs risk score

To begin with, we retrieved prognosis related DEGs by Kaplan–Meier analysis and univariate cox regression with Wald X^2^ test. In Kaplan–Meier analysis, patients were divided into two groups according to the median of expression level of DEGs. Their survivals were compared by log-rank test. The DEGs were considered significant when p-value of log-rank test is less than 0.05. Those DEGs was verified by univariate cox regression. Variables with Wald X^2^ test p-value less than 0.05 was selected. It is validated that 18 DEGs (EIF3C, WDR43, BICC1, HEATR1, PRPF40B, RBM4, RBM38, CLK3, EEF1D, SAMD4A, CTU1, RNASEH2A, HENMT1, ENOX1, FBXO17, SMG8, ZC3HAV1L, NUFIP1) were significantly in statistic with the overall survival of CC patients (shown in Fig. 5a). Moreover, these genes were applied to construct multivariate cox regression model, AIC value was used to sort the variable. Ten DEGs (EIF3C, WDR43, PRPF40B, RBM38, EEF1D, CTU1, RNASEH2A, HENMT1, ZC3HAV1L, NUFIP1) were preserved at last and regarded as prognosis related DEGs (Fig. 5b and Table 3).

**Table 3:** prognostic related gene sorted by multivariate cox regression

id	Coef.	HR	HR.95L	HR.95H	p value
EIF3C	0.68945	1.992619	0.862064	4.605842	0.106796
WDR43	− 0.85954	0.423357	0.224097	0.799793	0.00809
PRPF40B	1.29466	3.649756	1.710637	7.786992	0.000812
RBM38	− 0.62881	0.533228	0.370969	0.766457	0.000682
EEF1D	0.484649	1.623605	0.982808	2.682204	0.058457
CTU1	0.569026	1.766545	1.155554	2.700595	0.008599
RNASEH2A	− 0.74683	0.473867	0.298073	0.753339	0.001592
HENMT1	− 0.32793	0.720411	0.504799	1.028115	0.070737
ZC3HAV1L	0.538778	1.713911	1.058803	2.77435	0.028345
NUFIP1	0.991838	2.696187	1.344851	5.405375	0.005193

Four genes (WDR43, RBM38, RNASEH2A, HENMT1) played protective roles (HR < 1) among these ten DEGs. The other six genes (EIF3C, PRPF40B, EEF1D, CTU1, ZC3HAV1L, NUFIP1) were presented as risk factors for CC patients’ survival (HR > 1). Finally, the risk score was calculated according to the expression level of these ten genes ($\text{riskscore}={h}_{0}(\text{t})\text{exp}({\sum }_{j=1}^{n}{\text{Coef}}_{j}\times {\text{X}}_{j})$, n = 10, Coef_j_ is the coefficient of each DEG, X_j_ is the relative expression levels of each DEG, ${h}_{0}\left(\text{t}\right)$ is baseline risk function). The median risk score value was regarded as cutoff point to divide the CC patients into high risk group (n = 152) and low risk group (n = 152). The patients’ overall survival (OS) of high risk group is shorter than that of low risk group significantly (median time = 3.4 years vs. more than 8 years, log rank p < 0.001, Fig. 5c).

### Draw prognostic hazard curves

**Fig. 6:**
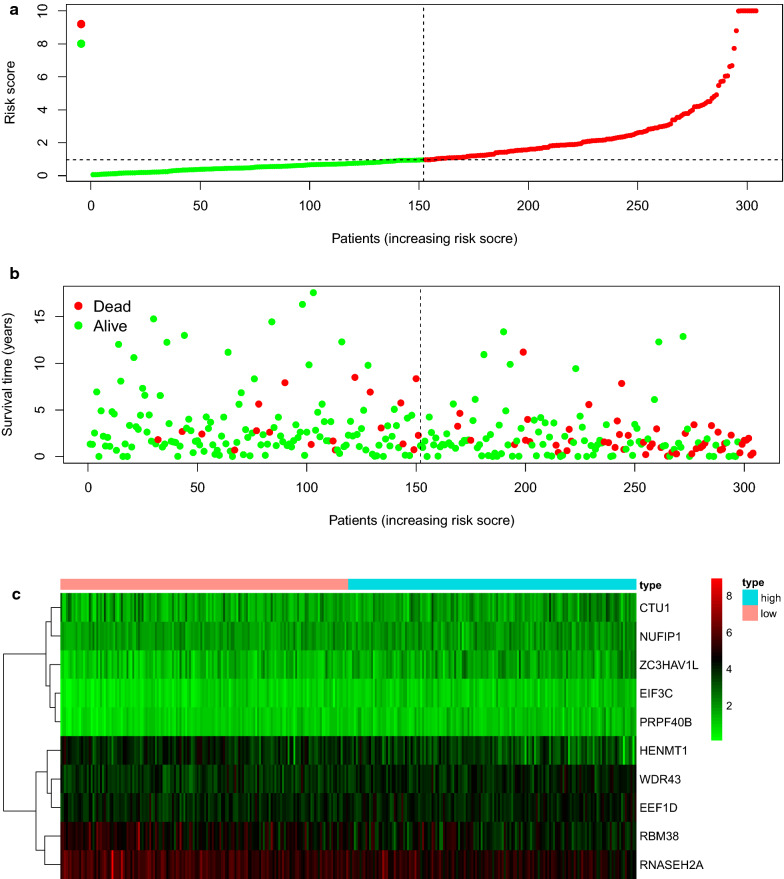
CC patients in high and low risk groups are stratified by risk score counted by expression level of RBPs for analyses. **a** Risk score scatter plot of high risk group patients and low risk group patients. Dead patients were presented as red dots. Alive patients were presented as green dots. The survival time of dead patients decreased with the ascend of risk score. **b** The individual inflection point of the risk score curve was displayed by dotted line. It shows that patients were divided into low-risk and high-risk groups by median of risk score. Red dots stood for patients with high risk. Green dots stood for patients with low risk. **c** Risk score heat map of ten DEGs. The expression level of ten DEGs increased with color varied from green to red

Prognostic hazard curves was drawn to evaluate the survival time for the patients. It is observed that the survival time diminished with the increasing of risk score for the dead patients (Fig. 6a, b). Furthermore, the quantity of patients alive decreased with the ascend of risk score for patients too. RNASEH2A was shown to be down-regulated in group with high-risk according to the risk heat map. Whereas, CTU1 was regarded as a tumor accelerating gene because it was up-regulated in high-risk group (Fig. 6c).

### Prognostic factors and prediction model for OS

**Fig. 7:**
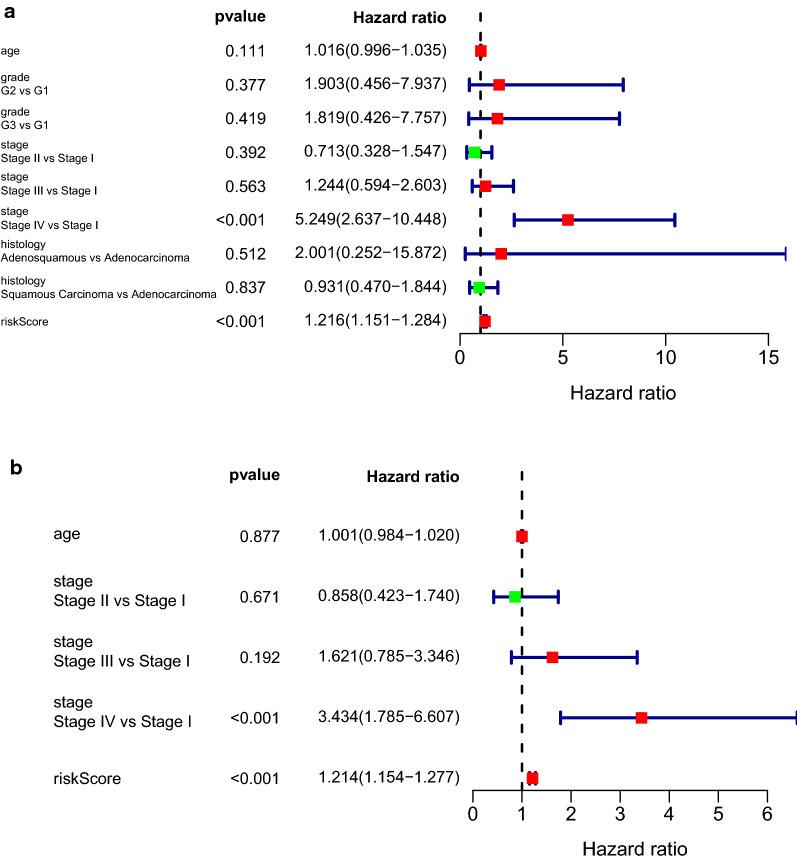
Forest plots of risk score and other clinical features. **a** Forest plot for risk score and clinical features in univariate cox proportional risk regression model. **b** Forest plot for risk score and clinical features in multivariate cox proportional risk regression model

The risk score and other clinical factors were combined to construct cox regression model. It is showed in univariate cox regression model that clinical stage and risk score were correlated with overall survival (OS) of CC patients (P < 0.001, P < 0.001, Fig. 7a). Multivariate cox regression validated that clinical stage (Stage IV vs Stage I HR = 3.434, P < 0.001) and risk score (HR = 1.214, P < 0.001) were independent risk factors for survival (Fig. 7b).

**Fig. 8:**
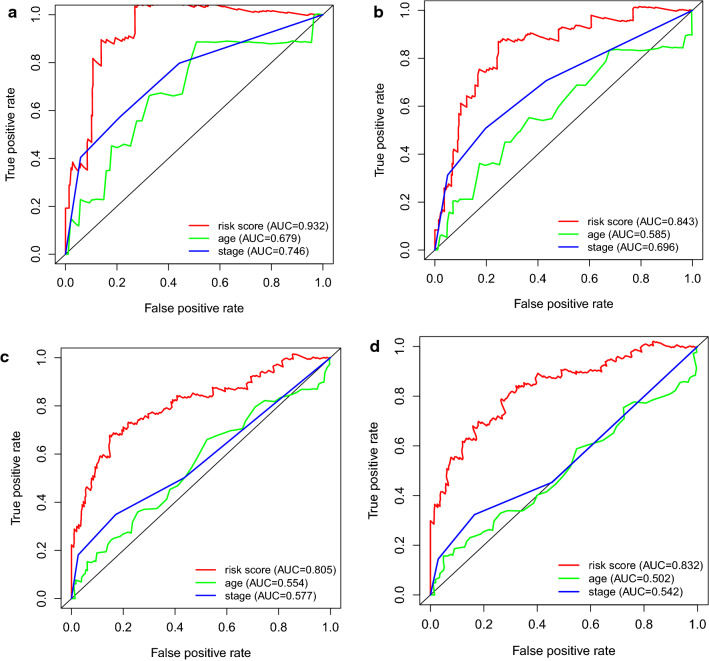
ROC curves for evaluating the discrimination of survival indicators **a** 0.5-year. **b** 1-year. **c** 3-year. **d** 5-year. AUC: area under curve. The discrimination feasibility increased with the ascending of AUC

For the sake of evaluating the discrimination of each predicting factors, ROC curves were constructed in 0.5-year, 1-year, 3-year and 5-year with the prediction factors (age, stage and risk score). Moreover, we assess the feasibility of discrimination of survival or dead patients using the area under curve (AUC) values. ROC curve reveals that the risk score showed a better ability to predict the survival of CC patients (AUC = 0.932, 0.843, 0.805, 0.832 for 0.5-year, 1-year, 3-year and 5-year) than other prediction factors (Fig. 8a–d).

### Analyze relationship between clinical features and DEGs predictor

**Fig. 9:**
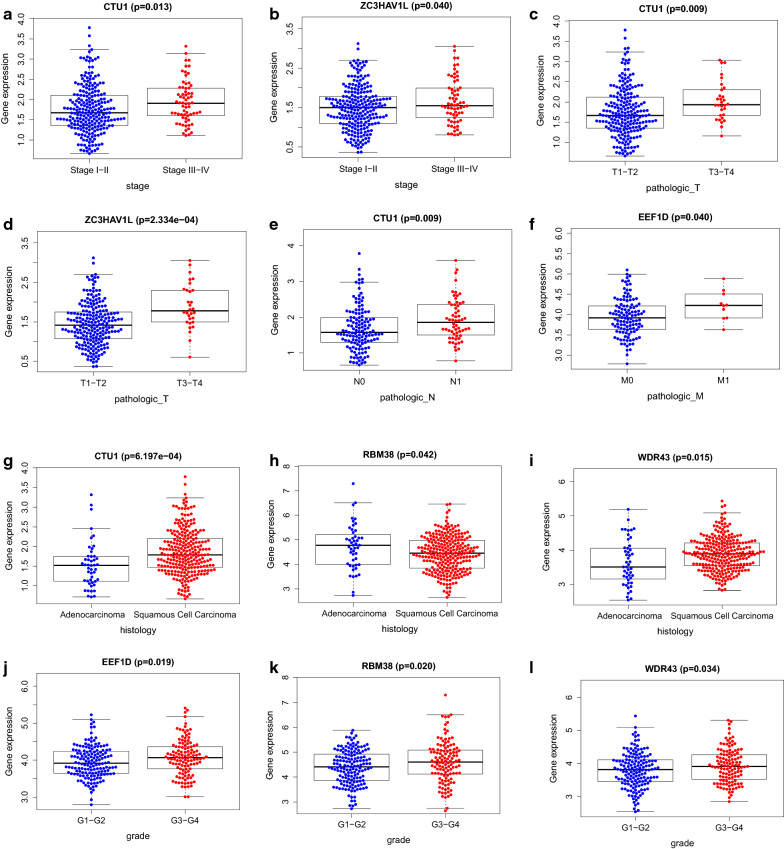
Box plot for displaying relationship between prognostic DEGs and clinical features (**a**–**j**)

The correlations between the ten prognostic DEGs and clinical features was evaluated by t-test or Kruskal–Wallis test depend on the quantity of categories of clinical features. It showed that the expression level of CTU1 and ZC3HAV1L were significantly different expressed in statistic with each clinical stage (P-values = 0.013 and 0.040 respectively) (Fig. 9a, b). Furthermore, the expression level of CTU1 and ZC3HAV1L were higher in the advanced T stage patients (P-values = 0.009 and < 0.001 respectively), implying their dangerous roles with the development of cervical cancer (Fig. 9c, d). The expression level of CTU1 was significantly associated with N stages which implying that its expression levels increased with progression of lymph node metastasis (Fig. 9e). The expression level of EEF1D increased with advanced M stage, which implied that it’s expression level may be correlated with the organ metastasis ability of cervical cancer (Fig. 9f). The expression level of CTU1, RBM38, WDR43 varies with different pathology of cervical cancer (Fig. 9g-i). In addition, the expression level of EEF1D, RBM38 and WDR43 ascended with higher tumor pathology grade (p-value = 0.019, 0.020, 0.034) (Fig. 9j–l).

### Establish and validate the nomogram

**Fig. 10:**
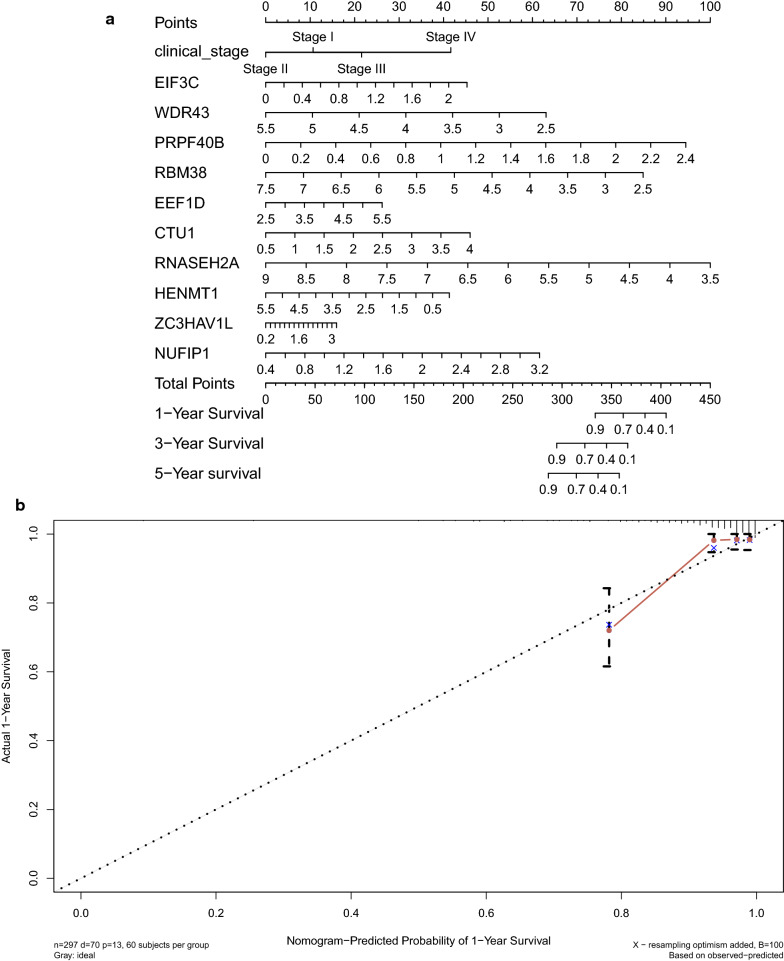

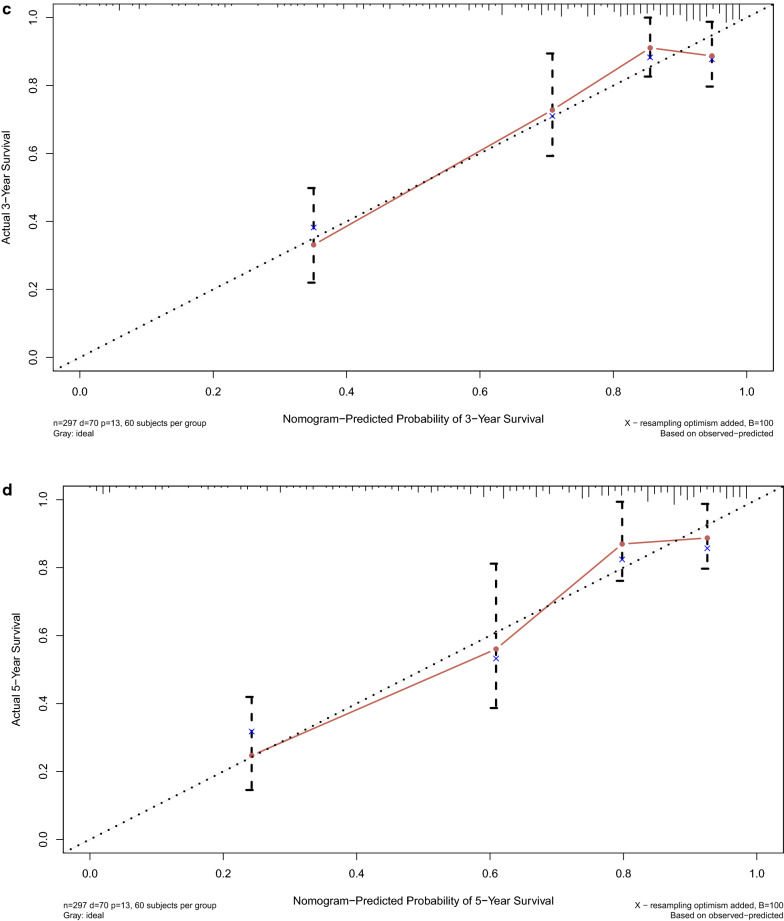
Prediction model constructed for CC patients. **a** Nomogram for CC with clinical stage and risk score which predict the survival of 1 year, 3 years and 5 years. **b** Calibration curves of the prognostic nomogram prediction in the 1-year. **c** Calibration curves of the prognostic nomogram prediction in the 3-year. **d** Calibration curves of the prognostic nomogram prediction in the 5-year

Three prognostic indicators including age, clinical stage and ten prognostic prediction RBPs were selected to establish the nomogram (Fig. 10a). The discrimination and calibration of nomogram was validated based on C-index and calibration curve. Analysis result revealed that the C-index of the constructed nomogram is 0.808 and the 1-year, 3-year and 5-year calibration curve in Fig. 10b–d demonstrated that the nomogram can partially predict the prognosis of CC patients.

### Enrichment analysis of immune cell and function

**Fig. 11:**
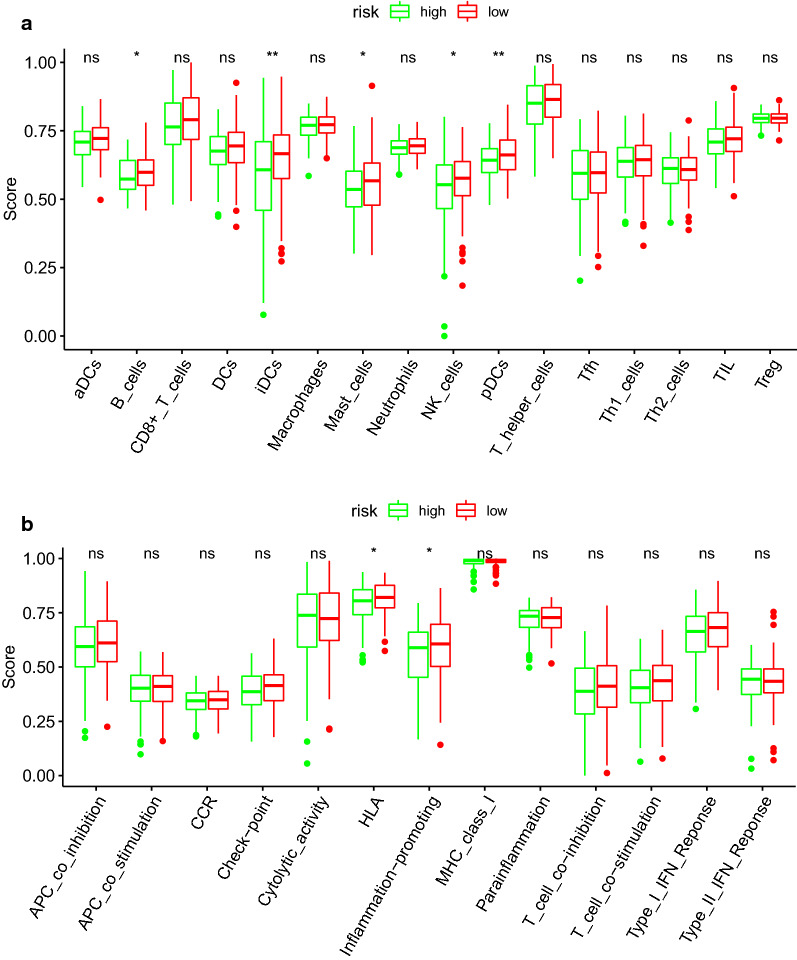

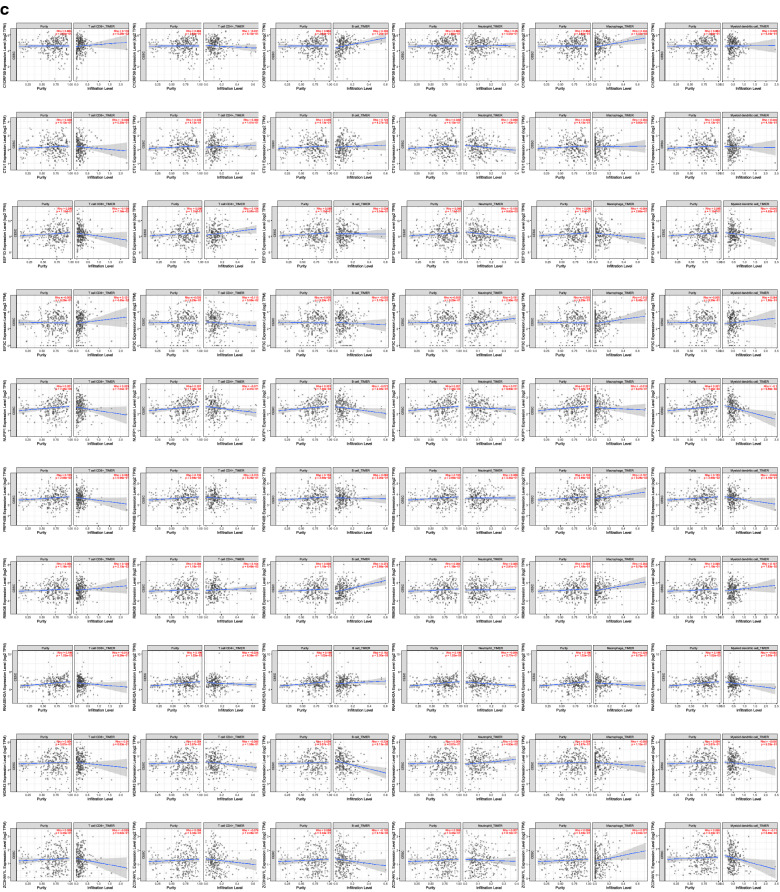
Box plot for ssGSEA immune score between the high and low risk groups categorized by median of risk score. **a** The scores of 16 immune cells. **b** The scores of 13 immune-related functions. DCs: dendritic cells; iDCs: immature DCs; pDCs: plasmacytoid dendritic cells; TIL: tumor infiltrating lymphocyte; CCR: cytokine-cytokine receptor; APC: antigen presenting cells. Adjusted P values were shown as: ns: not significant; *P < 0.05; **P < 0.01; ***P < 0.001. **c** Relationships between immune cells and prognosis related RNA binding proteins for cervical cancer patients. TPM: Transcripts Per Kilobase per Million mapped reads

SsGSEA R package was used to investigate the enrichment scores of 16 immune cell subpopulations and their 13 correlated immune functions. It is revealed that 5 kinds of immune cells (such as B cells, iDCs, mast cells, NK cells, pDCs) caught a lower score in high risk group than low risk group (Fig. 11a). What is more? The scores of the 2 types immune functions, such as HLA, Inflammation—promoting were significantly higher in low-risk group. Their enrichment scores suggested the immunological functions of high risk group should be injured more than low risk group classified by expression level of prognostic DEGs (Fig. 11b).

The correlation between the ten prognostic significant RBPs (WDR43, RBM38, RNASEH2A, HENMT1, EIF3C, PRPF40B, EEF1D, CTU1, ZC3HAV1L, NUFIP1) and the abundance of immune cells was analyzed by means of TIMER-2 database. The results showed that c1orf59 (alias HENMT1) is positively associated with B cell (p = 7.20e−07) and macrophage (p = 4.03e−05). CTU1 have positive correlation with B Cell (P = 4.27e−02). EEF1D has positive correlation with CD4+ T Cell (p = 8.57e−03) and negative correlation with Neutrophil (p = 9.62e−03). EIF3C has positive correlations with CD8+ T Cell (p = 4.45e−02) and Neutrophil (p = 2.96e−02). RBM38 has positive correlations with CD8+ T cell (p = 2.13e−02), B cell (p = 3.89e−06) and Macrophage (p = 6.76e−04). RNASEH2A has positive correlation with B cell (p = 2.30e−03). WDR43 has negative correlation with B cell (p = 6.13e−05) and positive correlation with Neutrophil (p = 4.93e−02) (Fig. 11c).

### RT-qPCR experiment validation

**Fig. 12:**
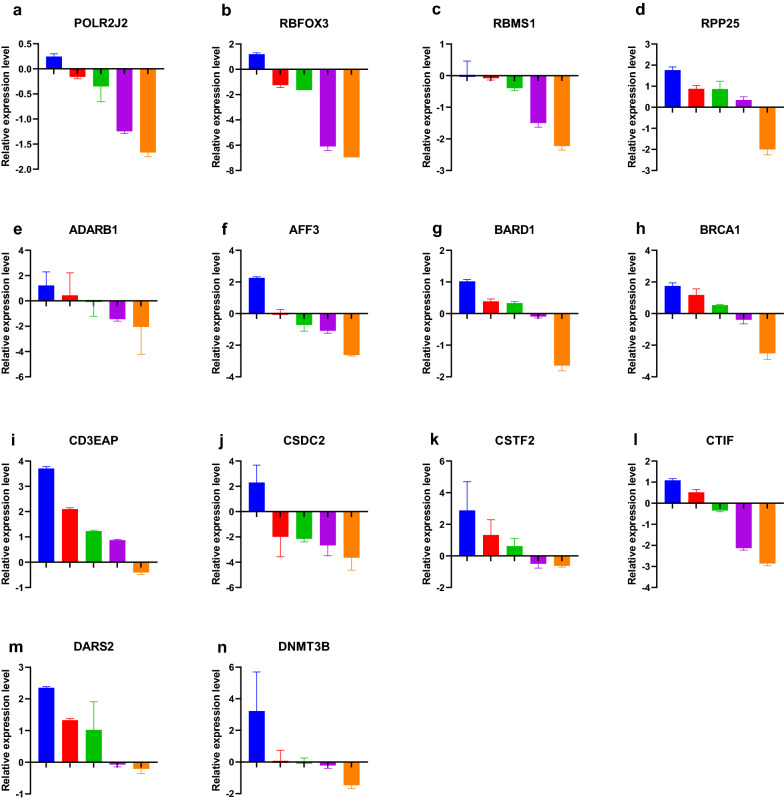
Relative expression level of differently expressed genes (RNA binding proteins) between normal tissues and cervical cancer tissues **a** POLR2J2, **b** RBFOX3, **c** RBMS1, **d** RPP25, **e** ADARB1, **f** AFF3, **g** BARD1, **h** BRCA1, **i** CD3EAP, **j** CSDC2, **k** CSTF2, **l** CTIF, **m** DARS2, **n** DNMT3B

Fourteen differently expressed genes (POLR2J2, RBFOX3, RBMS1, RPP25, ADARB1, AFF3, BARD1, BRCA1, CD3EAP, CSDC2, CSTF2, CTIF, DARS2, DNMT3B) was validated by RT-qPCR. The result shows that POLR2J2, RBFOX3, RBMS1, ADARB1, AFF3, CSDC2 and CTIF were down-regulated in most of cervical cancer tissues compared with corresponding normal cervix tissues. RPP25, BARD1, BRCA1, CD3EAP, CSTF2, DARS2 and DNMT3B was up-regulated in most of cervical cancer tissues compared with corresponding normal cervix tissues. These results required more validation in future by a larger scale clinical samples (Additional file 9: Table S9, Fig. 12).

### Kaplan–Meier analysis of prognosis significant RBPs

**Fig. 13:**
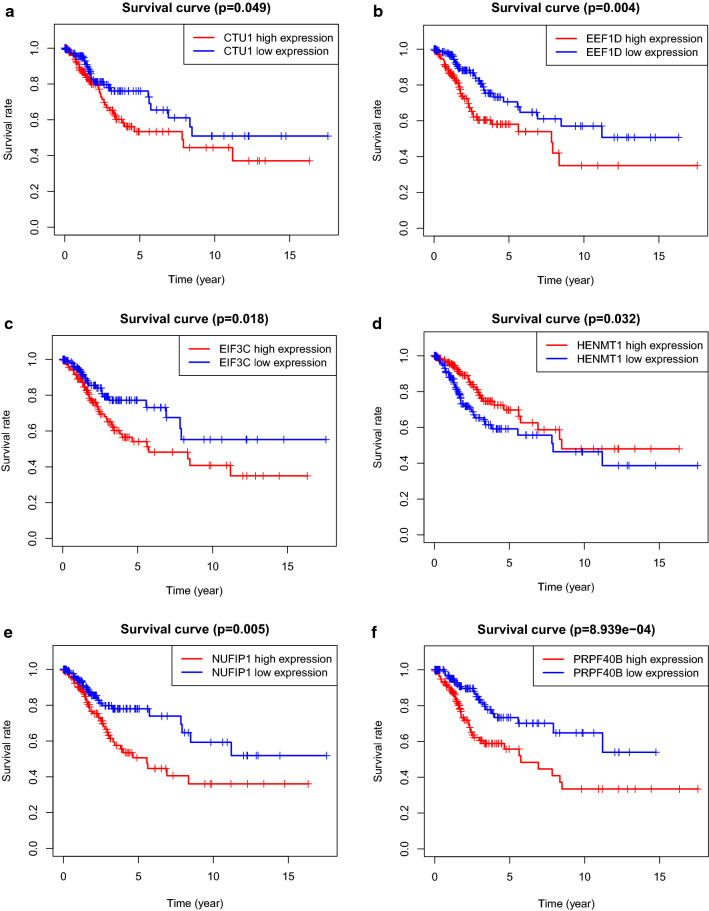

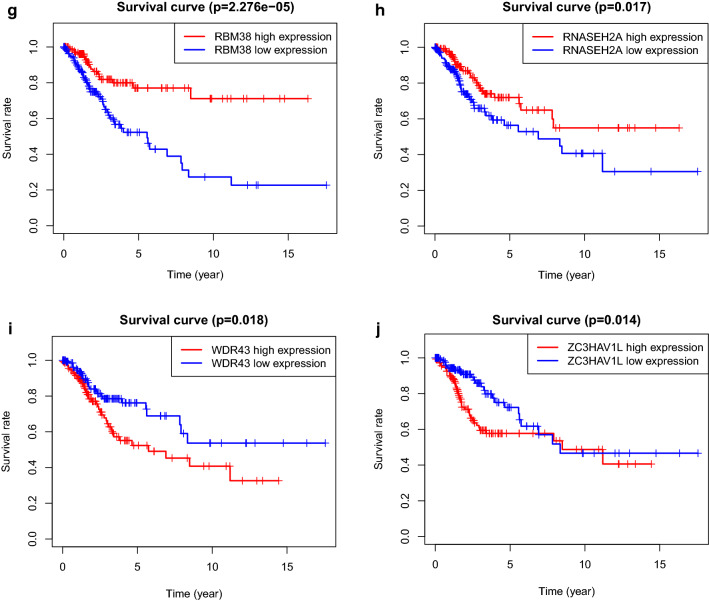
Kaplan-meier curve of prognostic significant RNA binding proteins in the prognosis model for cervical cancer patients. **a** CTU1, **b** EEF1D, **c** EIF3C, **d** HEMNT1, **e** NUFIP1, **f** PRPF40B, **g** RBM38, **h** RNASEH2A, **i** WDR43, **j** ZC3HAV1L

It is displayed in kaplan–Meier curves that higher expression level of EEF1D, CTU1, EIF3C, WDR43, NUFIP1, ZC3HAV1L and PRPF40B indicated a lower overall survival of cervical cancer patients. Nonetheless, higher expression level of HENMT1, RBM38 and RNASEH2A showed a better overall survival of cervical cancer patients. All of the overall survival differences between higher expression level group and lower expression level group of ten prognostic RBPs is significant in statistic which was verified by log-rank test (p < 0.05). (Fig. 13a–j).

### OncoPrint analysis in cBioPortal

**Fig. 14:**
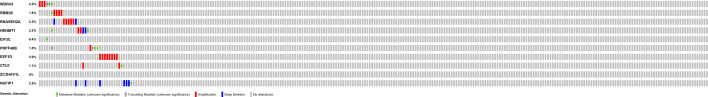
The variation of prognosis related RNA binding proteins’ expression in cancer tissues from CC patients were revealed by oncoprint analysis. Rows stand for genes and columns stand for CC patients. Missense mutation, truncating mutation, amplification and deep deletion are represented by glyphs and color codes

Gene expression variation of prognosis significant RNA binding proteins was explored by cBioportal tool with data of 178 CC tumors from patients in TCGA database. The clinical features of these patients were listed in Additional file 10: Table S10. OncoPrint analysis revealed that missense mutation was identified in WDR43, RBM38, HENMT1, EIF3C, PRPF40B, CTU1 and NUFIP1. Truncating mutation was discovered in WDR43 and PRPF40B. Amplification was seen in WDR43, RBM38, RNASEH2A, HENMT1, PRPF40B, EEF1D and CTU1. Deep deletion appears in RNASEH2A, HENMT1 and NUFIP1 (Fig. 14).

## Discussion

Nowadays, malignant tumor has become one of the greatest intimidation to human health, which has exceed the cardiovascular disease [25]. Cervical cancer has become the second most common malignancies among females all over the world [26]. Especially, in developing countries, where it is not popular for females to take part in cervical screening, cervical cancer posed a greater threat to woman than developed country [27]. Cervical intraepithelial neoplasia (CIN) was recognized as the precursor lesions for cervical cancer. Persistent infection of human papillomaviruses (HPVs) is one of majority reasons led to CIN [28]. The potential mechanism of CIN is assumed that the infection of virus alter gene transcription or affect the posttranscriptional regulation of message RNA. The possible process of posttranscriptional regulation included two categories. Firstly, microRNA is able to trigger degradation of the target message RNA by binding its 3’ untranslated regions (UTR) [29]. Secondly, RNA binding proteins involve in the process, editing, stability maintenance, transportation and translation of message RNA [10, 30]. Recently, microarray and RNA sequencing technologies have emerged as favorable tools for scientists to investigate the modification of cell’s gene or gene transcript in the development of cancer [31].

There are few prognosis predictive tools for clinical doctor to evaluate the survival of patients suffer from cervical cancer. The most widely used prognosis predictive tools is the international federation of gynecology and obstetrics (FIGO) staging system [17]. Nonetheless, its degree of accuracy remained to be improved. So, more prognosis markers are required for constructing a better prognosis model. It is popular for scholars to excavate potential cervical cancer prognosis related factors. Yang et al. discovered nine prognosis related genes which play significant role in cervical cancer immune environment [32]. Chen uncovered six immune related long noncoding RNA and constructed a prognostic predictive model for CC patient [33]. Qin recognized DSG2 as a biomarker which could predict the prognosis of early-stage cervical cancer [34]. Gao et al. reported a sample of CC prognosis biomarkers with four microRNA and seven hub genes [35]. The research of role that microRNAs play in the development of cervical cancer has been extended to exosomes [36]. They acted as signal transmission molecular performing genetic exchange between cells or took part in the development of chronic inflammation after HPV infection, which indicated their potential role as prognosis biomarker in cervical cancer [37]. In general, microRNA is a member of non-coding RNA family, whose role has been explained in many aspect of CC development such as lymphatic invasion, distant metastasis and angiogenesis [38–40]. Both of these study provided us with innovative vision of prognosis prediction of cervical cancer patients. Follow the research idea of former study, we innovative proposed a sample of RNA binding proteins involved in the progress of cervical cancer which might be valuable for CC diagnose or treatment.

In this study, we applied gene expression level data resourced from RNA sequencing technology and the clinical data of CC patients to explore the cervical cancer correlated RNA binding proteins. The RNA sequencing data of 306 cervical cancer tissues and 13 normal cervix tissues form GTEx and TCGA databases was integrated to analyze the expression profile of differently expressed RNA binding proteins (also called DEGs in this article, Differently expressed genes) in cervical cancer. 347 DEGs was retrieved by Wilcoxon sum-rank test, of which 177 DEGs were down regulated in tumor samples and 170 DEGs were up regulated in tumor samples. The functional enrichment analysis of GO and KEGG were performed for the downregulated and upregulated DEGs respectively. The PPI network was constructed for sorting the candidate genes of prognostic prediction model by STRING database. Moreover, this DEGs was screened by cox regression with Wald X^2^ test and Kaplan–Meier analysis with log-rank test. Among these DEGs, WDR43, RBM38, RNASEH2A and HENMT1 with HR < 1 played a protective role in survival. Other six genes (EIF3C, PRPF40B, EEF1D, CTU1, ZC3HAV1L, NUFIP1) were considered as risk factors with HR > 1. The nomogram was drawn to present the prognostic prediction model with FIGO stage and RBPs predictor. It was validated by C-index and calibration curve subsequently. In addition, the enrichment analysis of immune cell and function was performed by ssGSEA package in R software.

We investigated the biological functions of These DEGs by GO analysis. To begin with, the enrichment of cell components was located in the ribosome, cytoplasmic ribonucleoprotein granule and the ribonuclease. They play crucial roles in transmission of genetic information from DNA to protein. Protein was synthesized in ribosome by translating the coding information from RNA. The mutation of ribosomal protein may exert an influence on degradation of p53 protein which involved in the process of many kinds of cancer, such as endometrial cancer, T-cell acute lymphoblastic leukemia, chronic lymphocytic leukemia and colorectal cancer [41]. Many kinds of disease have been reported having something to do with RNA processing or RNA metabolism, which exerted influence on RNA translation [42–44]. The forming of ribonucleoprotein complexes has been recognized as the result of interaction of RNA and RBPs. They sustain the stability of target message RNAs, after which the efficiency of mRNA translation is promoted. For example, oncogenic RNA binding protein SRSF1 is reported to accelerate the proliferation of lung cancer cells by strengthening the message RNA stability of DNA ligase 1 [45]. What is more, ribonucleoprotein granule was discovered as a crucial region for protein synthesis. The development of cancer is affected by the modification of ribonucleoprotein, because of its significant role in RNA translation [41]. Moreover, the category of molecular function in GO analysis revealed the interactions of RNA and proteins such as RNA methyltransferase activity. RBPs have been discovered to bind with many kinds of RNA such as pre-mRNA, snRNA, tRNA and mRNA. The regulation of various enzyme was also displayed in GO analysis such as endoribonuclease, ribonuclease and nuclease. They are correlated to synthesis or repair of DNA and metabolism of RNA. For example, in the field of correlation between cervical cancer and RNA methylation. Pan et al. developed a prognostic prediction model for cervical cancer patients based on m6A RNA methylation regulator [46]. While, most of research concentrate on the methylation of protein or DNA such as the promoters of genes instead of RNA. The underlying mechanism of RNA methylation and CC remains to be revealed. Finally, in term of biological process category of GO analysis, differently expressed RBPs have something to do with the processing of both coding RNA and non-coding RNA such as rRNA and tRNA. Both of RNA splicing and metabolism were adjusted by these differently expressed RBPs. Our result was consistent with the consensus reached before. It is reported that RNA binding protein (RBP) quaking (QKI) was able to interact with the QKI response elements (QREs) in SLC26A4 gene introns, which lies in the 3’UTR (3’ untranslation region) of mRNA after transcription, thereby promoting circSLC26A4 biogenesis. CircSLC26A4 promotes the proliferation of cervical cancer cells in both vivo and vitro [47]. The RBP HuR was discovered to promote the growth of cervical cancer cells by interaction with the 3'UTR of RBP nucleolin (NCL) mRNA, which specifically promoted the translation of NCL without the alteration of NCL mRNA levels [48]. In other kind of cancer, RBP Musashi1 (Msi1) promoted the proliferation of colon cancer cells by target the 3’UTR of p21(cip1) [49]. Then, the items in KEGG pathway analysis suggested that the origination and development of cervical cancer is regulated by RBPs through mRNA surveillance pathway, RNA transport and RNA degradation. The underlying correlation between RBPs and signal pathways should be under research further.

The relationships between many RBPs and cancer has been reported by former studies which were consist with our study. We discovered that CTU1 is a risk factor for CC patients. It has been reported that CTU1/2, which is partner enzymes in U34 mcm^5^s^2^-tRNA modification, sustains metastasis and invasion of breast cancer [50]. Rapino et al. reported that the inhibition of CTU1 and proteins synergizing with it could kill melanoma cells [51]. Zhang et al. identified the copy number amplifications of CTU1 in 25% of myxopapillary ependymomas by means of whole exome sequencing [52]. CTU1 has been identified as one of prognostic predictors for prostate cancer and bladder cancer [53, 54]. We also identified NUFIP1 as a risk factor of CC patients. The forming of ETV6-NUFIP1 fusion gene has been reported as a potential cause of acute lymphoblastic leukemia in Mexico [55]. Deshpande et al. reported that NUFIP1 had something to do with genome stability maintenance [56] which may help cancer cells survive the pressure from environment. Mutated genes NUFIP1 had a higher level of expression in metastasis tumor than primary tumor in neuroblastoma indicating its oncogenic driver role [57]. However, the potential role of NUFIP1 in the process of cervical cancer development remains to be revealed.

The risk score calculated by expression level of DEGs was demonstrated to be a risk indicator. Patients in high risk group shows a significant lower survival than low risk group. ROC curve for risk factors suggested that risk score predicted the prognosis better than other factors which may be valuable in clinical application. It suggested that more precise therapeutic strategy should be applied to CC patients with higher risk score. At last the expression of each DEGs were analyzed with patients’ clinical features. CTU1 and ZC3HAV1L were significantly associated with clinic FIGO stage and T stage. Their oncogenic role was exposed gradually with the progress of clinical stage. What is more, the expression level of CTU1 increased with the N stage, which showed that it might promote the lymph node metastasis of cervical cancer. The expression level of EEF1D increased with M stage, which showed that it might had something to do with organ metastasis. Both of WDR43, CTU1 and RBM38 were correlated with pathology class. The expression level of EEF1D, RBM38 and WDR43 ascended with the progression of cancer pathology grade. This information may be a clue for further research about correlation between RNA binding proteins and clinical feature in cervical cancer.

Thanks to public database such as TCGA and GTEx, the correlation between prognosis of CC patients and RBPs was analyzed. The potential biological function of differently expressed RNA binding proteins was revealed by GO and KEGG enrichment analysis. A novel clinical prognosis prediction model was constructed for cervical cancer patients with RNA binding proteins. It is expected that this robust statistical support of CC could be used to help the RBPs researchers and clinical doctors in future. More clinical therapeutic schemes should be developed concentrating on RBPs genes in CC patients. There are some limits in this study. To begin with, the clinical stage, pathology grade and the treatment schemes downloaded from TCGA were incomplete. The HPV infection status of each patient was unknown. These deficiencies affected the accuracy of prediction model we constructed at last. Moreover, the potential mechanisms of how RBPs regulate the development of CC and their interaction relationship were remained to be explained. Finally, the nomogram has to be validated in a larger cohort, that will be helpful for further epidemical research. These deficiencies could be solved with a larger scale of clinical data appeared in future.

## Conclusion

347 DEGs (RNA binding proteins) were retrieved from gene expression profiles of cervical cancer and normal cervix tissues. Univariate and multivariate cox regression with Wald χ^2^ test were performed. Ten prognosis significant RBPs with potential diagnose and treatment value were presented by multivariate cox regression model optimized by AIC value. They were applied to construct nomogram which was expected to be validated in future. In addition, the biological functions of DEGs were analyzed by GO and KEGG analysis. Immune function of DEGs was analyzed by ssGSEA package in R software.

## Supplementary Information

**Additional file 1: Table S1.** Clinical pathological data of patients from TCGA database.

**Additional file 2: Table S2.** The annotation of 16 immune cells and 13 functions in ssGSEA

**Additional file 3: Table S3.** Clinical pathological data of own patients.

**Additional file 4: Table S4.** Differently expressed RNA binding proteins.

**Additional file 5: Table S5.** GO analysis of down-regulated RBPs.

**Additional file 6: Table S6.** KEGG analysis of down-regulated RBPs.

**Additional file 7: Table S7.** GO analysis of up-regulated RBPs.

**Additional file 8: Table S8.** KEGG analysis of up-regulated RBPs.

**Additional file 9: Table S9.** qPCR primary data.

**Additional file 10: Table S10**. Oncoprint analysis.

## Data Availability

All data were available in TCGA (https://portal.gdc.cancer.gov) and GTEx database (http://commonfund.nih.gov/GTEx/).

## Abbreviations

CC: Cervical cancer
RBPs: RNA binding proteins
OS: Overall survival
TCGA: The Cancer Genome Atlas
DEG: Differently expressed genes
FDR: False discovery rate
GO: Gene Ontology
BP: Biological process
CC: Cellular component
MF: Molecular function
KEGG: Kyoto Encyclopedia of Genes and Genomes
ROC: Receiver operating characteristic curve
AUC: Area under curve
HR: Hazard ratio
DCs: Dendritic cells
iDCs: Immature DCs
pDCs: Plasmacytoid dendritic cells
TIL: Tumor infiltrating lymphocyte
CCR: Cytokine–cytokine receptor
APC: Antigen presenting cells
Tfh: Follicular helper T cells
